# Perspectives on Artificial Intelligence in Nursing in Asia

**DOI:** 10.2196/55321

**Published:** 2024-06-19

**Authors:** Nada Lukkahatai, Gyumin Han

**Affiliations:** 1 School of Nursing Johns Hopkins University Baltimore, MD United States; 2 College of Nursing Research Institute of Nursing Science Pusan National University Busan Republic of Korea

**Keywords:** machine learning, ML, artificial intelligence, AI, algorithm, predictive model, predictive analytics, predictive system, practical model, deep learning, ChatGPT, chatbot, nursing, nurse, nursing education, personalized education, Asia

## Abstract

Artificial intelligence (AI) is reshaping health care, including nursing, across Asia, presenting opportunities to improve patient care and outcomes. This viewpoint presents our perspective and interpretation of the current AI landscape, acknowledging its evolution driven by enhanced processing capabilities, extensive data sets, and refined algorithms. Notable applications in countries such as Singapore, South Korea, Japan, and China showcase the integration of AI-powered technologies such as chatbots, virtual assistants, data mining, and automated risk assessment systems. This paper further explores the transformative impact of AI on nursing education, emphasizing personalized learning, adaptive approaches, and AI-enriched simulation tools, and discusses the opportunities and challenges of these developments. We argue for the harmonious coexistence of traditional nursing values with AI innovations, marking a significant stride toward a promising health care future in Asia.

## Introduction

Artificial intelligence (AI) is generally defined as a machine-based system that can make predictions, recommendations, or decisions to influence real or virtual environments based on human-defined objectives [1]. These systems—including branches such as robotics, machine learning, deep learning, and natural language processing—can imitate human cognitive functions such as reasoning, learning, and decision-making [2,3]. Over the years, AI has made significant advancements based on improved computer processing capabilities, access to large data sets for training, and algorithm designs [4]. AI-based technologies such as AI-powered decision support systems and AI-powered monitoring systems have been widely adopted by health care systems to improve patient care, enhance efficiency, and reduce costs [5,6]. Nurses are at the forefront of this revolution. AI can augment nurses’ abilities, thus improving patient outcomes and increasing clinicians’ and patients’ satisfaction [7-10].

The adoption of AI in nursing in Asia is varied but is a growing trend in the region. This viewpoint discusses our multifaceted perspectives on the use of AI in nursing practice and education, with a specific focus on Asian countries. It is important to note that this paper is not intended to be a systematic review of the topic but rather aims to highlight developing trends and prospects in the field.

## Applications of AI in Nursing

### Applications of AI in Nursing Practice and Research

The introduction of AI in nursing in Asia, as in other parts of the world, began to gain prominence in the late 20th century and continued to evolve over the years; however, the specific timeline for the first use of AI in nursing in Asia can vary depending on the region and health care institution (Table 1). Some Asian countries, particularly those with advanced health care systems and a strong focus on technology, may have adopted AI in nursing earlier than others. Regions such as Singapore, South Korea, Japan, India, and China have embraced AI-powered chatbots and virtual assistants, revolutionizing nursing practice and education, and addressing basic health queries [11-14].

As shown in Table 1, in practice and clinical research, Taiwan, South Korea, Japan, Singapore, and China have demonstrated significant advancements in the integration of AI. In Taiwan, data-mining techniques have significantly enhanced the prediction of nursing issues, while an electroencephalogram classification algorithm has greatly improved seizure monitoring. Hu et al [15] developed an inpatient pressure injury prediction model with an impressive 87.2% recall rate, benefiting high-risk patients. In South Korea, the automated sepsis risk assessment system (Auto-SepRAS) has excelled in categorizing sepsis risk, emphasizing its continuous monitoring value. AI-driven tools have effectively reduced hospital-acquired pressure ulcer rates and intensive care unit stays [16]. Additionally, recent studies in South Korea used machine learning–based analytical methods and natural language processing to accurately predict adverse drug reactions [17], pressure injury staging [18], and improve hospital data management capabilities [19]. Japan’s focus on advanced health care analytics is evident through the works of Nakatani et al [20] and Kawashima et al [21], which leveraged natural language processing and machine learning to predict hospital inpatient falls (area under the receiver operating characteristic curve of 0.834) and needs of cancer patients in palliative care, respectively. A study in China used machine learning–based analytical methods for the early detection of delirium in children with critical illnesses [22]. These examples illustrate the remarkable progress in AI integration in nursing across these Asian countries, contributing to improved patient care and safety.

The application of AI-based triage systems in health care facilities and AI-powered telemedicine can further improve access to health care for those who live in remote and conflict-affected areas [23-25]. A research group in Turkey used machine learning to assess pediatric pain to help address patient needs and experiences in clinical practice [26]. Despite the potential benefit of integrating AI into nursing practice to improve patient care and health care delivery, research in this area in developing countries is currently limited, and more studies are needed to explore the feasibility, acceptability, and effectiveness of AI-based solutions in real-world nursing settings.

A bibliometric analysis and science mapping study on AI research in nursing revealed that China has published 89 papers and that Japan and Korea each published 19 papers in this field among Asian countries [27]. In addition, a multinational collaboration network focusing on AI research in nursing has been formed, encompassing nations in Asia such as Japan, Thailand, India, China, Korea, and Singapore. However, the study lacked instances or a comprehensive examination of how Asian nations are implementing AI technology in the nursing domain, and it also failed to address the consequences of such technology on nursing practice and education. These limitations underscore the necessity for increased region-specific research and deliberate global cooperation to optimize the use of AI technology in the nursing domain within Asian nations.

**Table 1:** Examples of artificial intelligence (AI) in nursing practice and research across Asia.

Authors, year, and country	Study type	AI features	AI feature description	Application in nursing	Key findings
Aydın and Özyazıcıoğlu [26], 2023, Turkey	Primary research; observation study	ML^a^ (CNNs^b^)	Deep-learning models for visual data analysis, using layers to automatically learn and extract features from images	Postoperative pain assessment in children	ML closely matched children’s self-reported pain scores, demonstrating potential for clinical application
Back et al [16], 2016, South Korea	Primary research	AI-powered sepsis risk assessment system (Auto-SepRAS)	AI is used to analyze patient data and predict the likelihood of sepsis	Sepsis risk assessment	Auto-SepRAS demonstrated moderate predictive power for early sepsis identification in hospitalized patients
Hu et al [15], 2020, Taiwan	Primary research	ML (decision tree, logistic regression, random forest)	ML algorithms to make predictions and classifications based on data	Inpatient pressure injury prediction	The random forest model was the most accurate with key identified risk factors, including skin integrity and systolic blood pressure
Jeon et al [17], 2020, South Korea	Primary research	Temporal-difference method in reinforcement learning	Combining aspects of Monte Carlo methods and dynamic programming	ADRs^c^	Employing temporal-difference learning for analyzing ADRs from nursing notes offers promise for drug safety surveillance
Kawashima et al [21], 2024, Japan	Primary research	ML (XGBoost^d^)	ML algorithm based on gradient boosting used for classification and regression tasks	Specialist palliative care needs prediction	The predictive model showed potential to replace traditional screening tools, with high accuracy in identifying palliative care needs
Kim et al [18], 2023, South Korea	Primary research	CNN	Deep-learning models for visual data analysis	Pressure injury staging	The CNN model improved the accuracy of pressure injury staging decisions among health professionals
Khan et al [24], 2019, Bangladesh	Perspective	DHIS2^e^, EHR^f^, big data, AI, ML	The use of AI and ML in medical health record software	Health data warehouse, EHRs, workforce strategy	Bangladesh integrated fragmented health systems into a unified digital health platform, advancing national health care delivery and planning
Lei et al [22], 2023, China	Primary research	ML (XGBoost, logistic regression, random forest)	ML algorithms based on gradient boosting	Delirium prediction in pediatric intensive care	The XGBoost model was the best performer for early prediction of delirium in critically ill children
Nakatani et al [20], 2020, Japan	Primary research	NLP^g^ and ML	NLP focuses on the interaction between computers and human language; ML involves prediction algorithms	Predicting inpatient falls	High accuracy in predicting inpatient falls using nursing records with NLP and ML techniques
Shi et al [27], 2023, global (including Asia)	Bibliometric analysis	Various AI technologies	Not applicable	General nursing practice	Rapid growth in publications and citations in the field of AI in nursing, highlighting key areas such as nurse rostering, nursing diagnosis, decision support, and big data management; developed countries lead in publications and collaboration

^a^ML: machine learning.

^b^CNN: convolutional neural network.

^c^ADR: adverse drug reaction.

^d^XGBoost: extreme gradient boosting.

^e^DHIS2: District Health Information Software 2.

^f^EHR: electronic health record.

^g^NLP: natural language processing.

### Applications of AI in Nursing Education and Patient Support

As shown in Table 2, in nursing education, the integration of AI promises improved learning outcomes and an overall elevation in the quality of training by allowing personalized learning experiences [28-30]. Through intricate algorithms, educational content can be tailored to resonate with individual student needs, accounting for their unique strengths, weaknesses, and learning styles. This ensures content delivery in a manner most conducive to comprehension and retention. Adaptive learning allows students to assimilate knowledge at their own pace, optimizing their educational journey. Engaging and interactive modules instill genuine enthusiasm in learners, fostering an environment conducive to in-depth exploration and understanding [31,32]. Moreover, simulation tools enhanced by AI capabilities revolutionize hands-on nursing training, providing safe and controlled environments for students to practice and refine their skills. Real-time feedback within these simulations allows for immediate correction and learning that are instrumental in building clinical confidence [33-37]. The specific integration of AI in nursing education in Asia is varied by country and institution. Nevertheless, it is increasingly recognized as a valuable tool for improving the quality of education and for preparing nursing students for the complex health care environment.

While some countries such as India, Pakistan, Bangladesh, Turkey, and Afghanistan may face limited resources and infrastructure, several attempts have been made to develop low-cost, culturally tailored AI technologies to improve patient care, optimize workflow efficiency, and enhance clinical decision-making (Table 2). Examples of such AI applications in these countries include the implementation of AI-powered chatbots for patient education and support [23,38].

**Table 2:** Examples of artificial intelligence (AI) in nursing education and patient support across Asia.

Authors, year, and country		Study type	AI features	AI feature description	Application in nursing	Key findings
**Nurse education and provider training**						
	Chen et al [31], 2022, China	Primary research	Chatbot	AI program designed to simulate conversation with human users	History-taking instruction program	Identified a need for chatbot-based history-taking instruction to provide more practice and feedback opportunities
	Liao et al [8], 2015, Taiwan	Primary research; case study	BPN^a^, ANFIS^b^	BPN is a machine-learning model that learns by adjusting its connections based on errors. ANFIS combines neural networks and fuzzy logic to learn and make decisions from data.	Support decision-making in nursing; generate nursing diagnoses	AI can assist in accurately generating nursing diagnoses with an agreement rate of up to 87% between system suggestions and nurse-made diagnoses.
	Liaw et al [37], 2023, Singapore	Primary research; RCT^c^	AI in virtual reality simulation	Using AI to create realistic and interactive virtual environments, enhancing the user’s experience	Sepsis care and interprofessional communication training	Virtual reality simulations with AI-powered doctors were effective for sepsis team training without inferior outcomes
	Castonguay and Lovis [30], 2023, Canada	Reflection article	ChatGPT	A language model developed by OpenAI designed to understand and generate human-like text based on the input it receives	Nursing education, research, and practice	ChatGPT could revolutionize nursing education by supporting students’ learning, improving digital literacy, and facilitating critical thinking. Despite potential biases and limitations, it can serve as a tool for research, teaching, and summarizing complex documents. Its integration requires collaboration to establish competencies and ethical guidelines for AI use in nursing
**Patient education and support**						
	Cheng et al [32], 2023, Taiwan	Primary research; interventional study	AI chatbot	AI program designed to simulate conversation with human users	Peritoneal dialysis care	The AI chatbot significantly improved patient satisfaction and reduced infection rates
	Castonguay et al [29], 2023, global (including Asia)	Comparative study	AI	A technology that enables machines to mimic human intelligence, allowing them to learn, reason, and make decisions	AI maturity in health care systems	Most OECD^d^ countries are at the emerging level of AI maturity in health care. Only the United States and the United Kingdom have achieved the integrated ecosystem level, indicating mature, collaborative AI use in health care. The study underscores the need for adaptable, context-specific AI strategies for health care across different countries.
	Castonguay et al [28], 2024, global (including Asia)	Editorial	AI language models	Systems that use AI to understand and generate human-like text based on the data they have been trained on	Health care digitalization	AI language models have significant potential to improve decision-making and patient engagement in health care. Challenges include ensuring reliability, transparency, and ethical use. The new journal section aims to explore, showcase, and address these challenges.
	Park et al [19], 2024, South Korea	Primary research	NLP^e^	Focuses on the interaction between computers and human language	Patient interaction, health records management	Enhanced communication and improved data management capabilities
	Simsek-Cetinkaya and Karaveli Cakir [38], 2023, Turkey	Primary research; interventional design	Interactive screen-based simulation	A digital tool that lets users engage with simulated scenarios on a screen, allowing them to practice skills or experience situations	Breast self-examination training	AI simulation increased student satisfaction but was less effective than standard simulation for teaching skills
	Wang et al [23], 2022, India	Primary research; interventional study	AI chatbot	AI program designed to simulate conversation with human users	Sexual and reproductive health education	The chatbot engaged users, particularly young men, providing a private space for discussing sensitive health topics

^a^BPN: back-propagation neural network.

^b^ANFIS: adaptive neuro-fuzzy inference system.

^c^RCT: randomized controlled trial.

^d^OECD: Organisation for Economic Co-operation and Development.

^e^NLP: natural language processing.

## Challenges of AI in Nursing Practice in Asia

While AI promises to revolutionize health care in Asia, it also presents several challenges. A primary concern is the lack of consistent standards and regulations for AI tools. This lack of standardization can lead to patient safety issues, particularly if devices from different manufacturers do not integrate smoothly or yield inconsistent results [39]. Biases embedded within AI algorithms are another significant concern. If the training data for these algorithms do not represent diverse populations, the AI systems might produce discriminatory or unequal outcomes. Such biases could exacerbate existing health care disparities or introduce new ones, thus challenging the equity and fairness of care delivery [40].

Ethical challenges—particularly related to data privacy and informed consent—are also paramount. As the health care industry increasingly relies on vast data sets, ensuring data security and transparent usage is crucial. Addressing patient autonomy and consent for data usage is of utmost importance. Moreover, disparities in resources and infrastructure across Asia’s vast landscape can hinder uniform AI adoption. While urban health care centers readily adopt AI, rural areas may face challenges such as outdated equipment or inconsistent internet connectivity. Finally, the integration of AI necessitates an educational shift for nurses, emphasizing a balance between clinical knowledge and technological skills [41-44].

The use of an AI-powered chatbot in nursing education presents some challenges. One of the foremost challenges is the need for adequate infrastructure and resources to implement AI technologies effectively. Many educational institutions may face financial constraints or lack the technical infrastructure required for seamless AI integration. Additionally, there are concerns related to the appropriate and ethical use of AI in education, including issues of data privacy, bias in AI algorithms, and transparency in decision-making processes. Educators and institutions must also address the potential resistance to change among faculty members and students who may be unfamiliar with AI-based tools and systems. Balancing the human touch and critical thinking skills that are so intrinsic to nursing with the technological advancements in AI poses another challenge, as this requires a thoughtful approach to curriculum design and the development of AI-enhanced educational content that aligns with nursing practice.

Furthermore, while some AI-powered dialogue systems (eg, ChatGPT, Microsoft Bing AI, Google Gemini) have the potential to enhance nursing education by providing instant access to information, facilitating virtual simulations, and offering personalized learning experiences, there are concerns regarding their potential misuse. Growing concerns are related to students becoming overly dependent on AI-generated responses along with the risk of misinformation or inaccurate guidance because these systems lack access to up-to-date evidence-based knowledge or clinical expertise [29,34,44]. In nursing education, where critical thinking, empathy, and clinical judgment are vital, overreliance on AI could inadvertently undermine these essential skills.

Introducing AI integration in nursing in Asia presents several challenges that are rooted in resource constraints, technological infrastructure disparities, data privacy concerns, cultural acceptance, resistance to change, education and training gaps, the need for ethical and legal frameworks, language diversity, and integration with existing health care systems. Resource limitations often hinder investments in AI technology and staff training, while disparities in technological infrastructure and connectivity across regions can hinder access to advanced AI tools. Developing robust data-protection regulations and cybersecurity measures is essential to address privacy concerns. Overcoming cultural and traditional health care practices, as well as ensuring that AI is embraced by both health care providers and patients, requires a thoughtful approach. Education and training are crucial, as health care professionals need specialized training to effectively use AI tools. Developing ethical guidelines and legal frameworks, as well as addressing the issues related to language diversity and the seamless integration of AI with existing systems, are complex but necessary steps to ensure successful AI adoption in nursing across Asia. Despite these challenges, many Asian countries are actively working to overcome these barriers, recognizing the potential benefits of AI in nursing for improving patient care, increasing efficiency, and enhancing health care outcomes.

## Summary and Prospects

In summary, the advent of AI is indicating a significant transformation in the field of nursing across Asia. Embracing these innovations necessitates the recognition of the enduring importance of the human touch and empathy within the profession. When effectively integrated, AI can complement and coexist with the core values of traditional nursing, paving the way for a harmonious and promising future in health care. Despite our interpretation of current evidence and perspective of the role of AI in nursing practice and education in Asia, this is not a systematic review. The limitation of this viewpoint is that the potential lack of comprehensive data specific to AI use in nursing across all Asian countries, the depth of analysis and generalizability of findings, and cultural and contextual differences across countries may not be fully captured to shape our perspectives. These limitations highlight the need for a follow-up systematic review paper and further research.

## Abbreviations

AI: artificial intelligence
